# Efficacy of combined doxycycline and intense pulsed light therapy for the management of intractable recurrent chalazion

**DOI:** 10.3389/fmed.2024.1454049

**Published:** 2024-09-30

**Authors:** Hyeon-Jeong Yoon, Ja Young Moon, Kyung Chul Yoon

**Affiliations:** Department of Ophthalmology, Chonnam National University Medical School and Hospital, Gwangju, Republic of Korea

**Keywords:** recurrent chalazion, meibomian gland, blepharitis, doxycycline, intense pulse light therapy

## Abstract

Management of chalazion, characterized by noninfectious chronic granulomatous inflammation of the meibomian glands (MGs), remains challenging in ophthalmic practice, particularly because of its propensity for recurrence even after surgical intervention. This study aimed to evaluate the combined efficacy of doxycycline and intense pulsed light (IPL) therapy for treating intractable recurrent chalazion, focusing on reducing recurrence rates and improving MG status. This retrospective study included patients with intractable recurrent chalazion refractory to conventional treatments including surgical intervention and antibiotics. All patients were treated with a combination of doxycycline and IPL therapy. Clinical assessments included best-corrected visual activity, intraocular pressure, and MG evaluations using slit-lamp biomicroscopy and Keratograph 5 M topography. The study included 12 patients (5 male and 7 female) with an average age of 36.3 years. Treatment outcomes were categorized as ‘success’ (83.3%), ‘stationary’ (8.3%), and ‘failure’ (8.3%). Significant improvements were noted in the MG expression scores (*p* = 0.038), MG quality scores (*p* = 0.045), and lid margin telangiectasia scores (*p* = 0.002). In addition, significant improvement in meiboscores was observed (*p* = 0.002). The combination of doxycycline and IPL therapy demonstrated promising efficacy in treating intractable recurrent chalazion, with significant improvements in MG function and reduced recurrence rates. This treatment approach offers a viable alternative for patients with recurrent chalazions, particularly for those who are unresponsive to conventional treatments.

## 1 Introduction

Management of chalazion, characterized by noninfectious chronic granulomatous inflammation of the meibomian glands (MGs), remains challenging in ophthalmic practice, particularly because of its propensity for recurrence even after surgical intervention (1, 2). In most cases, chalazion is self-limiting or resolves with conventional ophthalmic treatments such as warm compression, antibiotic ointments, steroid injections, or incision and curettage (I&C) of the lesion (1, 2). However, conventional treatments often fail to provide a lasting solution, and many patients experience repeated episodes (3, 4). Frequent recurrence not only provokes to patient discomfort, but also raises concerns about long-term ocular health, including the risk of MG loss and subsequent complications such as dry eye (5, 6).

Recent research has focused on nonsurgical treatments to address the underlying causes of chalazion recurrence (4, 5, 7). Intense pulsed light (IPL) therapy, originally developed for dermatological conditions, has shown considerable promise in managing MG dysfunction (MGD), a condition associated with chalazion (8, 9). IPL was effective in reducing the chalazion size, alleviating discomfort, and enhancing MG function and could be a novel approach for curbing the cycle of recurrence (4, 5, 7). Moreover, the efficacy of synergistic application of IPL with MG expression (MGX) in patients with refractory MGD suggests a broader applicability in cases of recurrent chalazion (4, 7).

In addition to IPL, several studies have assessed the role of systemic antibiotics in conditions associated with MGD such as ocular acne rosacea and blepharo-keratoconjunctivitis (10–12). Doxycycline, a tetracycline antibiotic, has antimicrobial property and inhibits matrix metalloproteinases that degrease connective tissues (13). Moreover, the efficacy of doxycycline in pediatric patients with ocular conditions similar to chalazion supports the potential of systemic antibiotics for managing recurrent chalazions (14). Application of doxycycline was intended to manage the underlying inflammatory processes that exacerbate chalazion recurrence.

This study aimed to explore the efficacy of combined doxycycline and IPL therapy for the treatment of intractable recurrent chalazion, with a focus on reducing recurrence rates and improving MG status.

## 2 Materials and methods

This study was approved by Institutional Review Board of Chonnam National University Hospital (CNUH-2024-125). The study protocol adhered to the guidelines of the Declaration of Helsinki.

### 2.1 Study population

This retrospective case series evaluated the efficacy of combined doxycycline and IPL therapy in 12 patients with intractable recurrent chalazions. The inclusion criteria were as follows (1): Patients with intractable recurrent chalazion who did not respond to conventional treatment (including lid hygiene, warm compression, topical or systemic antibiotics, and I&C) for at least 3 months, who were treated with combined doxycycline and IPL therapy between March 2022 and December 2023 at Chonnam National University Hospital (2); age ≥ 18 years (3); Patients with Fitzpatrick skin types 1–4 with no contraindication for IPL therapy; and (4) patients without gut problems and contraindication for oral doxycycline treatment. The exclusion criteria were as follows (1): presence of active skin lesions, skin cancer, active ocular infection, or inflammatory disease, which might affect the outcome, and (2) follow-up duration <2 months.

### 2.2 Treatment protocol

**Figure 1:**
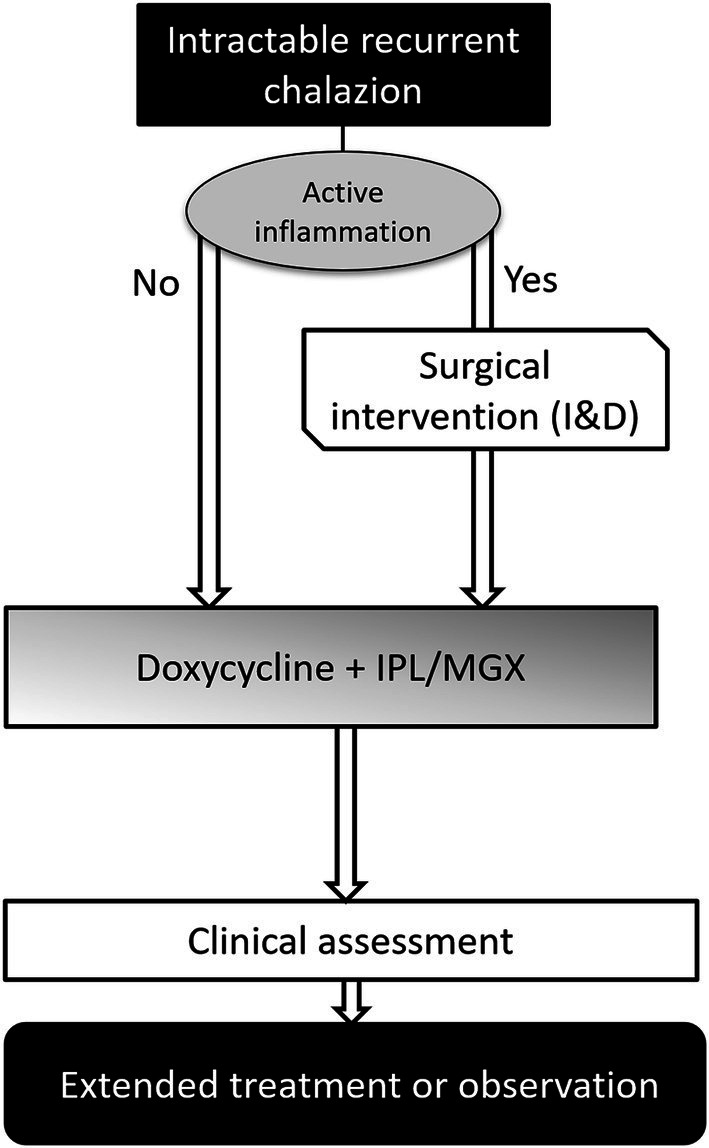
Flowchart of clinical treatment protocol in this study.

The treatment protocol is presented in Figure 1. At the first visit, an ocular assessment was performed to check for active inflammation (painful swelling, pus discharge, etc.). If patients had severe lid inflammation, surgical intervention, specifically incision and curettage (I&C), was prioritized to treatment protocol. Patients with controlled inflammation underwent combined IPL therapy and MGX and were prescribed doxycycline (Young Poong Pharm. Co., Ltd., Incheon, Korea) at a dose of 100 mg, twice daily. The standard duration of doxycycline therapy was three weeks. However, depending on individual patient responses and conditions, this duration could be extended, with the maximum duration capped at three months. The treatment protocol was adjusted based on individual responses.

Prior to IPL therapy, patients were classified according to their Fitzpatrick skin type and advised to remove any makeup (15). During treatment, sonographic gel was applied to the eyelids, and a Jaeger lid plate (Suzhou Mingren Medical Equipment, Suzhou, China) was positioned in the conjunctival sac to protect the cornea and sclera. IPL therapy was administered using an M22^®^ device (Lumenis, Dreieich, Germany) equipped with an acne filter. The treatment involved irradiating the lower eyelids eight times and the upper eyelids three times using a cylindrical 6 mm sapphire cooling tip at an intensity of 15 J/cm (16, 17). MGX therapy was administered after topical anesthesia with proparacaine eye drops (Paracaine; Hanmi Pharm, Seoul, Korea). A consistent force was applied to the medial, central, and lateral portions of both the upper and lower eyelids using Arita forceps (Katena Products, Parsippany-Troy Hills, NJ, United States). These therapies were administered over three sessions at three-week intervals, with adjustments to two or four sessions, depending on the patient’s condition. All procedures were performed by an experienced corneal specialist (H. J. Y.).

### 2.3 Data collection and clinical assessment

Patient information (age, sex, comorbidities, disease duration, number of recurrences, and ocular history) were retrospectively collected from the electronic medical records. In cases where both eyes were affected, the more severe eye was included in the analysis. Best-corrected visual activity, intraocular pressure, and slit-lamp biomicroscopic examinations were performed before and after treatment. In addition, we checked for typical findings of blepharitis such as redness, swelling, and dandruff-like scales at the base of the eyelashes. MG quality, MG expressibility, and lid margin telangiectasia score were graded from 0 to 3, as previously described (18). Subsequently, the meiboscore was evaluated using a Keratograph 5 M (Oculus Optikgerӓte GmbH, Wetzlar, Germany). The meiboscore was graded, to assess the extent of meibomian gland loss, ranging from grade 0 (no loss) to grade 3 (more than two-thirds of the gland area lost) (18).

In this study, the primary outcomes of the study were recurrence rate and improvement in MG status. MG status was measured at one month following the final IPL session, and recurrence was tracked throughout the follow-up period. Treatment outcomes were classified into three categories: ‘success,’ ‘stationary,’ and ‘failure.’ Success was defined as no recurrence of chalazion, accompanied by significant improvement in meibomian gland parameters and lid status. Stationary was defined as no recurrence but no significant improvement in lid status. Failure was defined as the recurrence of chalazion with active inflammation.

### 2.4 Statistical analysis

Statistical analysis was performed using SPSS version 22.0 for Windows (IBM Corp., Armonk NY, United States). Data are presented as mean ± standard deviation. The Wilcoxon signed-rank test was used to compare the changes in variables before and after treatment. Statistical significance was set at *p* < 0.05.

## 3 Results

Among the 12 patients included in the study, 5 were male and 7 were female. The mean age was 36.3 ± 14.3 years (range, 20–64 years). The average duration of chalaziosis was 11.3 ± 7.5 months (range, 7–18 months). All patients presented with blepharitis. Of the 12 eyes evaluated, 6, 3, and 3 had chalazions on the upper lid, lower lid, and both the upper and lower lids, respectively. All 12 patients (100%) were practicing lid hygiene and continuing the use of topical antibiotics at the time of their visit, and 6 patients (50%) had previously taken oral systemic antibiotics, which were discontinued prior to their visit to our clinic. Additionally, 11 patients had a history of surgical intervention prior to the current presentation (Table 1).

**Table 1:** Demographics and baseline characteristics of 12 patients with intractable recurrent chalazion.

Characteristics	*N* = 12
Age (mean ± SD), years	36.3 ± 14.3
Sex (M:F)	5: 7
Visual acuity (LogMAR)	0.02 ± 0.04
Intraocular pressure (mmHg)	16.2 ± 2.37
Duration of chalaziosis (mean ± SD), months	11.3 ± 7.5
Chalazion location	
Upper lid	6 (50.0%)
Lower lid	3 (25.0%)
Upper and lower lid	3 (25.0%)
Number of recurrences (mean ± SD)	6.4 ± 3.2
Pre-existing blepharitis	12 (100%)
Previous treatment	
Lid hygiene	12 (100%)
Topical antibiotics	12 (100%)
Excision	11 (91.7%)

Among the included patients, seven commenced IPL sessions within 1–2 weeks of initiating doxycycline treatment, whereas five patients underwent surgical intervention before starting IPL therapy owing to the persistence of active inflammation. Before treatment, patients experienced 6.4 ± 3.2 times recurrences over the course of 11.3 ± 7.5 months. After the combined doxycycline and IPL treatment, only one patient (8.3%) experiencing a recurrence during the follow-up period. Regarding outcomes, 10 (83.3%), 1 (8.3%),’ and 1 (8.3%) were classified as success, stationary, and failure, respectively. One patient with treatment failure was diagnosed with concurrent skin rosacea and experienced two recurrent episodes. One patient reported gastric discomfort as a side effect (Table 2).

**Table 2:** Demographics and baseline characteristics of patients with intractable recurrent chalazion.

Patient number	Age (yrs)	Sex	Comorbidity	Eyelid	Duration of chalaziosis (months)	Number of recurrences	Previous treatment	Initial surgical intervention (yes/no)	Doxycycline (weeks)	IPL session (times)	Follow up duration (months)	Outcome	Recurrence after final treatment (times)	Adverse effects
1	32	M		All	13	12	I&C, anti**	Yes	8	3	5	Success	0	None
2	27	M		RUL	25	6	I&C, anti**	No	3	2	6	Stationary	0	None
3	50	F		LUL, LLL	6	5	I&C, anti**	Yes	4	4	7	Success	0	None
4	25	F		RUL, RLL	9	5	I&C, anti**	No	3	2	8	Success	0	None
5	46	M		RLL, LUL	6	4	I&C, anti*	Yes	3	2	6	Success	0	None
6	26	F		RUL, LUL	21	13	I&C, anti*	No	3	4	6	Success	0	None
7	21	F	Skin rosacea	RUL, LUL	12	6	I&C, anti*	Yes	12	2	6	Fail	2	None
8	24	F		RUL, LUL	4	5	I&C, anti*	Yes	9	3	7	Success	0	Gastric discomfort
9	64	M		RLL LLL	7	4	I&C, anti*	No	6	4	7	Success	0	None
10	47	M		RUL	24	10	I&C, anti*	No	3	2	4	Success	0	None
11	54	F		LLL	4	3	anti**	No	3	2	4	Success	0	None
12	20	F		RUL, LUL	5	4	I&C, anti**	No	3	3	6	Success	0	None

IPL, intense pulse light; RUL = Right upper lid; LUL = Left upper lid; RLL = Right lower lid; LLL, left lower lid; I&C=Incision and curettage; anti*, antibiotic (topical), anti**, antibiotic (topical & systemic).

**Figure 2:**
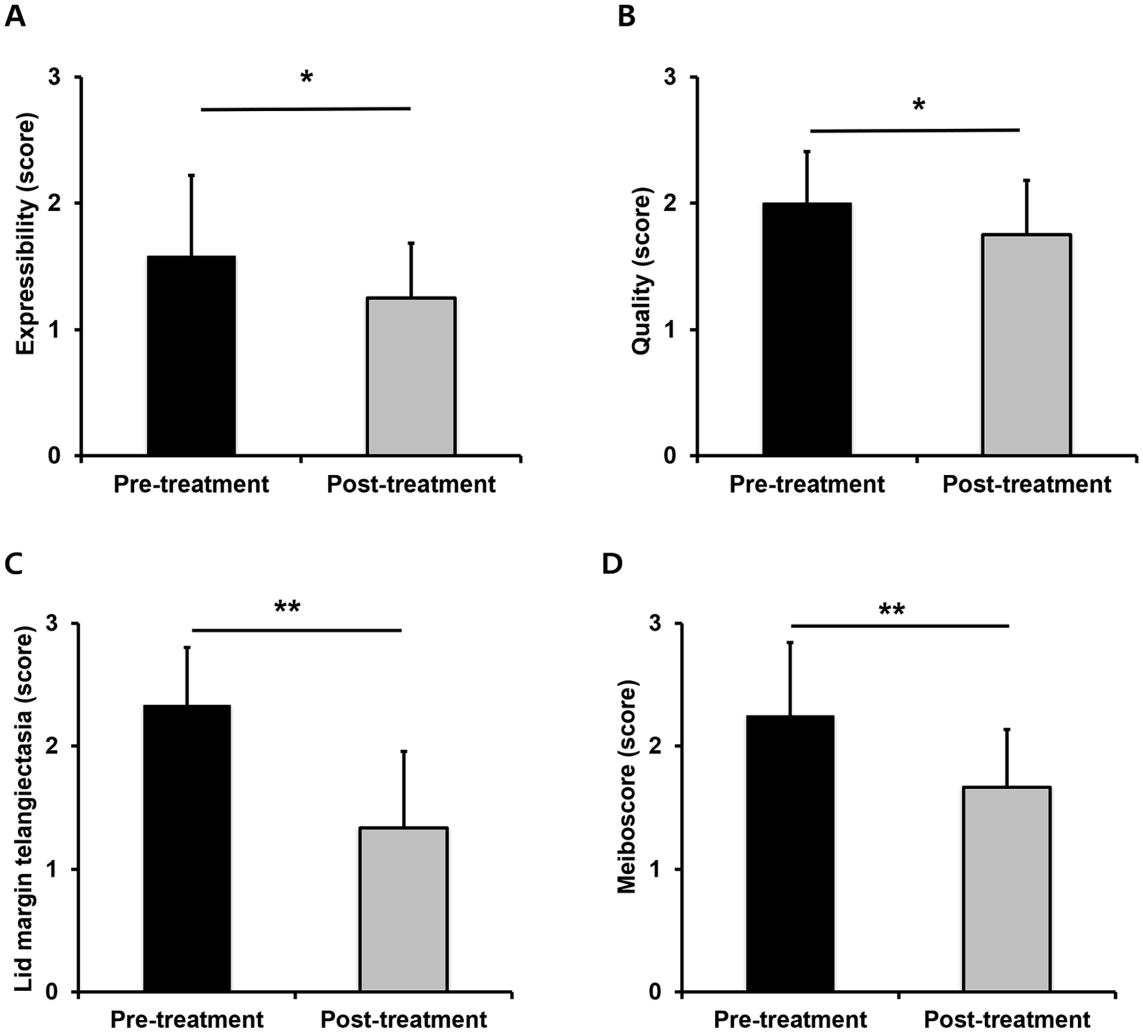
Changes in meibomian gland parameters between baseline and 1 month after the final IPL-MGX treatment session; expressibility **(A)**, quality **(B)**, telangiectasia **(C)**, and meiboscore **(D)**; *p < 0.05 or **p < 0.01 is considered statistically significant.

**Figure 3:**
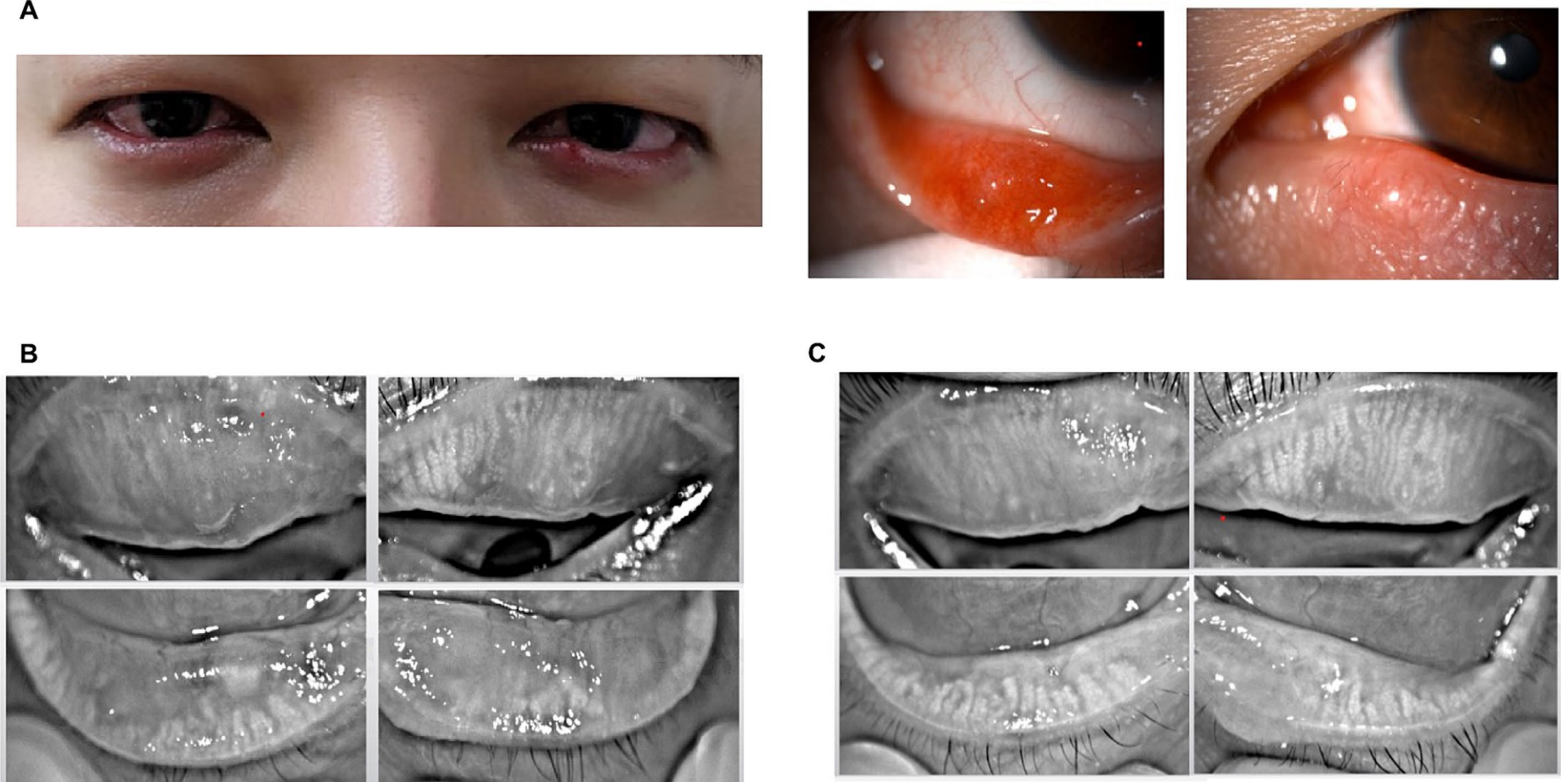
Representative cases with severe recurrent chalazion; slit-lamp photography **(A)** and meibography before treatment **(B)**, and meibography after final IPL-MGX treatment session **(C)**.

MG expressibility scores showed significant improvement from a mean of 1.58 ± 0.64 before treatment to 1.25 ± 0.43 post-treatment (*p* = 0.038, Figure 2A). Similarly, MG quality scores improved significantly from 2.00 ± 0.40 before treatment to 1.75 ± 0.43 after treatment (*p* = 0.045, Figure 2B). Furthermore, lid margin telangiectasia scores decreased from 2.33 ± 0.47 before treatment to 1.33 ± 0.62 after treatment (*p* = 0.002, Figure 2C), and meiboscores significantly improved from 2.25 ± 0.59 before treatment to 1.67 ± 0.47 after treatment (*p* = 0.002; Figure 2D). Representative figures is presented in Figure 3.

Pre-treatment visual acuity was 0.02 ± 0.04 LogMAR and IOP was 16.2 ± 2.37 mmHg, while post-treatment values were 0.01 ± 0.03 LogMAR and 16.5 ± 1.84 mmHg, showing no significant differences.

## 4 Discussion

Chalazion is a common eyelid disease characterized by chronic granulomatous inflammation of the MGs (1, 2). However, preventing recurrence and reducing treatment rates remains challenging (19). Recurrent chalazion can cause cosmetic disfigurement of the eyelid, and the accompanying ocular inflammation may cause ocular irritation, discomfort, visual impairment due to mechanical ptosis, or corneal astigmatism (4, 20). Some patients with multiple recurrent chalazions have a decreased quality of life (4). Intractable recurrent chalazions are usually associated with chronic blepharitis, meibomitis, or acne rosacea (1, 4). Therefore, in addition to treating chalazion, management of the morphological aspects of MGD is important (4). Multiple factors are involved in the pathogenesis of chalazion, including seborrheic, hormonal, immunological, infectious (e.g., *Staphylococcus aureus*, *Cutibaterium acnes*, and demodicosis), and dysmetabolic (e.g., vitamin A deficiency or diabetes) factors (1, 6, 19, 21–23).

Our findings showed that patients with intractable recurrent chalazion refractory to conventional treatment, including surgical intervention, can be effectively treated with a combination of doxycycline and IPL therapy. Clinical assessments associated with MGs (expressibility, quality, lid margin telangiectasia, and meiboscores) significantly improved after combination treatment. Particularly, lid margin telangiectasia and meiboscores showed significant improvement with *p*-value of 0.002 and 0.008, respectively. These parameters likely reflect active inflammation of the MGs in the eyelids.

IPL therapy, often used for dermatological conditions, is effective for treating ocular conditions such as dry eye disease related to MGD and ocular rosacea, which are closely related to chalazion. Several studies have (4, 5, 7) shown that IPL therapy combined with MGX is an effective, safe, and noninvasive treatment method for decreasing the recurrence rate of chalaziosis by promoting MG function (4, 5, 7). IPL can create a positive feedback loop that controls the inflammatory process by upregulating anti-inflammatory substances and/or downregulating pro-inflammatory substances (24, 25). Furthermore, upregulation of the skin temperature by approximately 70°C induces thermal changes in the MG including meibum liquefaction and MG unplugging (5, 26). Additionally, IPL reduces the loading of various microbes that mediate blepharitis (e.g., *Demodex folliculum*) (5).

Although antibiotics are generally not indicated for chalazion, doxycycline was chosen in this study due to its anti-inflammatory properties to exacerbate chalazion recurrence, especially in cases where severe blepharitis were present (14). Tetracycline antibiotics are effective for treating blepharitis and acne rosacea by decreasing bacterial lipase production (11, 27). Therefore, they can lower the concentration of free fatty acids and decrease the production of microbial inflammatory matrix metalloproteinases that degrease connective tissues (4, 14). The incidence of dyspepsia with doxycycline is lower than that with other tetracyclines; therefore, it provides better gastric comfort (28). Several studies have shown that doxycycline improves tear-film parameters by improving MG function (10, 12, 29). The use of doxycycline is based on its potential for managing chronic chalazion, particularly in cases of underlying blepharitis (10, 12, 29). Combining IPL with doxycycline could offer a synergistic effect; IPL mainly addresses the physical aspects of MGD and ocular-surface inflammation, whereas doxycycline targets microbial components and inflammatory pathways. This dual approach could lead to more comprehensive management of chronic recurrent chalazion and related ocular surface diseases.

In this study, only 1 out of 12 patients (8.3%) experienced recurrence. All patients in our research continued to practice lid hygiene and use topical antibiotics. Additionally, our cohort included 11 patients who had already undergone surgery yet continued to suffer from recurrent episodes over 3 times. Despite these challenging conditions, our recurrence rate was similar to the 11.4% recurrence rate observed in studies utilizing IPL therapy alone (4, 5, 7). Therefore, this combination therapy demonstrates superior effectiveness compared to IPL monotherapy.

This study had several limitations. First, this was a retrospective case series with a small sample size, which may introduce selection bias and limit the generalizability of the findings. Second, the number of IPL sessions and the duration of doxycycline treatment were not identical, and the decision criteria were relatively subjective. Third, there is a possibility that missing data in patient follow-ups may have influenced the accuracy of long-term recurrence assessments. Further studies with larger patient cohorts and prospective designs are required to reach definitive conclusions. Future studies should focus on the long-term outcomes and effectiveness of this combination therapy in a broader range of patients with meibomitis-related ocular conditions.

In conclusion, the combination of doxycycline and IPL therapy, which integrates the anti-inflammatory action of doxycycline with the targeted therapeutic effects of IPL, offers a promising and less invasive alternative for patients who continue to experience post-surgery chalazion recurrence. Combination therapy significantly improved MG function and reduced recurrence rates. This approach is expected to not only address immediate symptoms but also target the underlying causes of the condition, potentially improving long-term ocular health.

## Data Availability

The original contributions presented in the study are included in the article/supplementary material, further inquiries can be directed to the corresponding author.
